# Health-related quality of life associates with change in FEV_1_ in COPD: results from the COSYCONET cohort

**DOI:** 10.1186/s12890-020-1147-5

**Published:** 2020-05-29

**Authors:** Johanna I. Lutter, Rudolf A. Jörres, Kathrin Kahnert, Larissa Schwarzkopf, Michael Studnicka, Stefan Karrasch, Holger Schulz, Claus F. Vogelmeier, Rolf Holle, for the COSYCONET Study Group, Stefan Andreas, Robert Bals, Jürgen Behr, Kathrin Kahnert, Burkhard Bewig, Roland Buhl, Ralf Ewert, Beate Stubbe, Joachim H. Ficker, Manfred Gogol, Christian Grohé, Rainer Hauck, Matthias Held, Berthold Jany, Markus Henke, Felix Herth, Gerd Höffken, Hugo A. Katus, Anne-Marie Kirsten, Henrik Watz, Rembert Koczulla, Klaus Kenn, Juliane Kronsbein, Cornelia Kropf-Sanchen, Christoph Lange, Peter Zabel, Michael Pfeifer, Winfried J. Randerath, Werner Seeger, Michael Studnicka, Christian Taube, Helmut Teschler, Hartmut Timmermann, J. Christian Virchow, Claus Vogelmeier, Ulrich Wagner, Tobias Welte, Hubert Wirtz

**Affiliations:** 1 Institute of Health Economics and Health Care Management, Helmholtz Zentrum München, GmbH – German Research Center for Environmental Health, Comprehensive Pneumology Center Munich (CPC-M), Member of the German Center for Lung Research (DZL), Ingolstaedter Landstr. 1, 85764 Neuherberg, Germany; 2 Institute and Outpatient Clinic for Occupational, Social and Environmental Medicine, University Hospital, LMU Munich, Comprehensive Pneumology Center Munich (CPC-M), Member of the German Center for Lung Research (DZL), Ziemssenstr. 1, 80336 Munich, Germany; 3 Department of Internal Medicine V, University of Munich (LMU), Comprehensive Pneumology Center, Member of the German Center for Lung Research, Ziemssenstr. 1, 80336 Munich, Germany; 4 Department of Pneumology, Paracelsus Medical University Salzburg, Universitätsklinikum Salzburg, Müllner Hauptstrasse 48, 5020 Salzburg, Austria; 5 Institute of Epidemiology, Helmholtz Zentrum München (GmbH) – German Research Center for Environmental Health, Comprehensive Pneumology Center Munich (CPC-M), Member of the German Center for Lung Research (DZL), Ingolstaedter Landstr. 1, 85764 Neuherberg, Germany; 6 Department of Medicine, Pulmonary and Critical Care Medicine, University Medical Center Giessen and Marburg, Philipps-University Marburg, Member of the German Center for Lung Research (DZL), Baldingerstrasse, 35043 Marburg, Germany; 7 Institute for Medical Informatics, Biometry and Epidemiology, University Hospital Ludwig-Maximilians-University Munich (LMU), Marchioninistr. 15, 81377 Munich, Germany

**Keywords:** COPD, Cohort, Longitudinal, Patient reported outcome, Health status, Physical activity

## Abstract

**Background:**

Forced expiratory volume in one second (FEV_1_) characterizes the pathophysiology of COPD and different trajectories of FEV_1_ decline have been observed in patients with COPD (e.g. gradual or episodic). There is limited information about the development of patient-reported health-related quality of life (HRQL) over the full range of the natural history of COPD. We examined the longitudinal association between change in FEV_1_ and change in disease-specific and generic HRQL.

**Methods:**

We analysed data of 1734 patients with COPD participating in the COSYCONET cohort with up to 3 years of follow-up. Patients completed the Saint George’s Respiratory Questionnaire (SGRQ) and the EQ-5D Visual Analog Scale (EQ VAS). Change score models were used to investigate the relationship between HRQL and FEV_1_ and to calculate mean changes in HRQL per FEV_1_ change categories [decrease (≤ − 100 ml), no change, increase (≥ 100 ml)] after 3 years. Applying hierarchical linear models (HLM), we estimated the cross-sectional between-subject difference and the longitudinal within-subject change of HRQL as related to a FEV_1_ difference or change.

**Results:**

We observed a statistically significant deterioration in SGRQ (total score + 1.3 units) after 3 years, which was completely driven by the activity component (+ 4 units). No significant change was found for the generic EQ VAS. Over the same period, 58% of patients experienced a decrease in FEV_1_, 28% were recorded as no change in FEV_1_, and 13% experienced an increase. The relationship between HRQL and FEV_1_ was found to be approximately linear with decrease in FEV_1_ being statistically significantly associated with a deterioration in SGRQ (+ 3.20 units). Increase in FEV_1_ was associated with improvements in SGRQ (− 3.81 units). The associations between change in FEV_1_ and the EQ VAS were similar. Results of the HLMs were consistent and highly statistically significant, indicating cross-sectional and longitudinal associations. The largest estimates were found for the association between FEV_1_ and the SGRQ activity domain.

**Conclusions:**

Difference and change in FEV_1_ over time correlate with difference and change in disease-specific and generic HRQL. We conclude, that deterioration of HRQL should induce timely re-examination of physical status and lung function and possibly reassessment of therapeutic regimes.

**Trial registration:**

NCT01245933. Date of registration: 18 November 2010.

## Background

Chronic obstructive pulmonary disease (COPD) is defined by the presence of post-bronchodilator airways obstruction, respiratory symptoms such as breathlessness, cough, and sputum production and a history of exposure to inhalational injury [1]. Patients with COPD experience an accelerated decline in FEV_1_ compared to healthy never smoking individuals, where a decline of about 20 ml per years was shown [2]. However, the natural history of COPD is not always characterized by a gradual accelerate decline but can also present as episodic accelerated decline of FEV_1_. Here, episodes of deteriorated and improved lung function mark the overall downward trajectory of lung function over time [3]. Accordingly, patients with declining or rapidly declining FEV_1_ but also patients with stable or even improved FEV_1_ over time have been identified in large COPD cohorts [4–6].

While measures like FEV_1_ and blood gases reflect the pathophysiology of COPD, measures of health-related quality of life (HRQL) reflect the patient’s perspective of his/her disease. They are meaningful instruments to monitor the course of COPD as they cover the severity of symptoms, the impact of the disease on daily life and have also been found to predict mortality [7–9]. The longitudinal association between change in FEV_1_ and change in HRQL is not fully understood. Estimates based on RCTs and only few observational studies range from only a weak correlation [10] to strong correlations [11–13] and often focus on one direction of FEV_1_ change– i.e. decrease only [14] or increase only [11, 13]. Furthermore, the transferability of findings from RCTs to routine care is limited, because of highly selected patient samples.

In summary, there is limited information about the development of HRQL over the full range of the natural history of COPD, which includes FEV_1_ decrease in the context of exacerbations, FEV_1_ increase as a consequence of treatment, as well as unchanged FEV_1_. We therefore analysed data from a large, real-world observational cohort of COPD patients followed for 3 years, with the aim to analyse and possibly quantify the association between longitudinal FEV_1_ change and change in generic and disease-specific HRQL.

## Methods

### Study design and study population

Between September 2010 and December 2013, the prospective, multicentre COSYCONET (“German COPD and Systemic Consequences – Comorbidities Network”) study recruited 2741 participants in 31 study centres across Germany and re-examinations took place after 18- and 36-months. Briefly, baseline inclusion criteria of COSYCONET were age ≥ 40 years and a physician’s diagnosis of COPD. Detailed information about the inclusion and exclusion criteria and the recruitment process are available elsewhere [15].

**Fig. 1:**
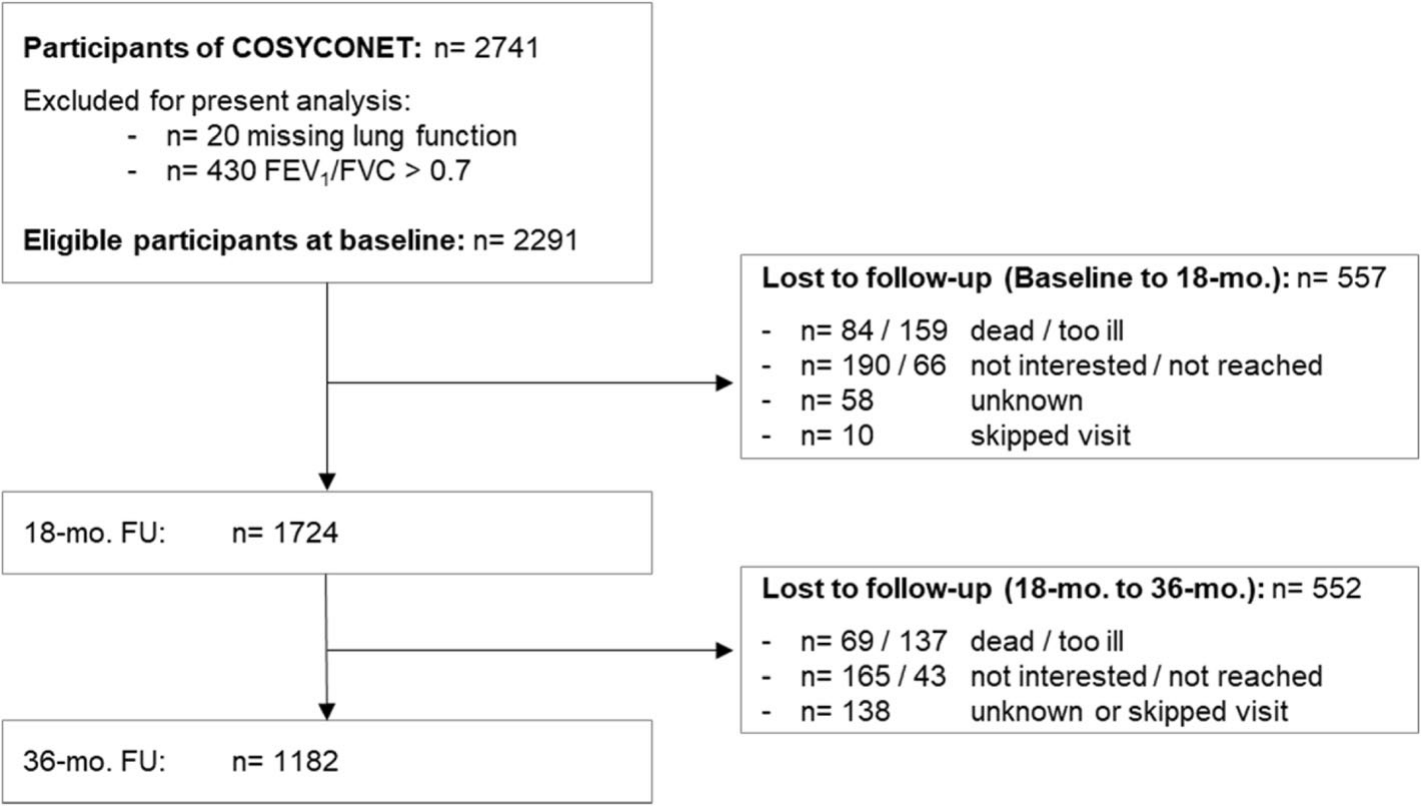
Overview of the study population. Abbreviations: mo. = months; FU = follow-up

For the present analysis, we excluded patients with (a) missing FEV_1_ values at baseline, (b) FEV_1_/FVC ≥ 0.7 at baseline, and (c) no further study participation after baseline. Patients with alpha-1-antiythrypsin deficiency were not excluded, since their HRQL was found to be comparable to patients without the immune deficiency in a cross-sectional analysis [16]. An overview of the study population is given in Fig. 1.

### COPD definition and HRQL assessment

Participants underwent standardized post-bronchodilator spirometry at each visit. GOLD grades 1–4 were assigned at baseline based on FEV_1_ predicted, whereby reference values were taken from the Global Lung Initiative [17]. For the stratified analysis, GOLD grades were further aggregated in two groups (GOLD 1/2 and 3/4) because of limited numbers of patients in GOLD grades 1 and 4 (each less than 10% of the total sample).

At each visit, HRQL was assessed using two self-administered questionnaires: the generic 3-level version of Euro-Qol 5D (EQ-5D-3 L) and the disease-specific Saint George’s Respiratory Questionnaire for COPD (SGRQ) [18, 19]. The generic instrument EQ-5D is designed to assess HRQL regardless of a specific disease and consists of two parts, the descriptive section and the valuation section. For the present analysis, we used the descriptive section only, i.e. the Visual Analogue Scale (EQ VAS), since this descriptive section was found to better discriminate between COPD grades compared to the EQ-5D valuation section. Furthermore, the VAS was preferred as a simple measure of generic HRQL since the EQ-5D utility index requires a country-dependent tariff and is less sensitive due to its skewed distribution [20]. When using the EQ VAS, participants value their current health status on a scale between 0 (worst possible) and 100 (best imaginable) and a 6.9 units change has been proposed as the minimal clinically important difference [21]. To assess disease-specific HRQL, we used the SGRQ in its COPD specific version. This questionnaire consists of 40 questions related to three components of HRQL (symptoms, activity, and impacts). The total score ranges between 0 and 100 with higher values indicating worse HRQL. Its reliability, validity and responsiveness has been demonstrated in patients with COPD and a 4 units change is considered to indicate the minimal clinical important difference [22].

### Assessment of covariates

Age, sex, education, and smoking status were assessed in standardized interviews complemented by self-administered questionnaires. Body mass index (BMI) was calculated based on measured height and weight. Information on 33 comorbid conditions was obtained by asking “Has a physician ever diagnosed one of the following diseases?”. This information was summarised into a single count indicating the number of comorbidities (range 0–33) at each visit. This approach has been previously proven to be a sufficient proxy for total comorbidity burden [23]. Again based on self-reports the history of exacerbations was defined according to GOLD guidelines as no exacerbation, mild, moderate, or severe exacerbation. For each patient, only the most severe exacerbation that occurred in the 12 months preceding the respective study visit was coded. In this way, we attempted to minimize a potential recall bias especially with regard to an underestimation of lighter events. In case of missing values, we imputed the most frequent category or the mean value for continuous data. Considering all three visits and > 4500 observations, a total of only 25 values were imputed for the covariates.

### Statistical analysis

Since loss of lung function and HRQL are both dependent on disease severity, patient characteristics including FEV_1_ and measures of HRQL at baseline and all analyses are reported stratified by GOLD grade (1/2 vs. 3/4) [4, 24]. For 1182 patients with participation in the 3 year follow-up, change in FEV_1_ and HRQL over 3 years was evaluated based on t-tests for paired data. To investigate the association between FEV_1_ and HRQL over time, two statistical approaches were employed: change score analysis and hierarchical linear models. All models were adjusted for age, sex, BMI, education, smoking status, number of comorbidities, and exacerbation history.

#### Change score analyses

First, using ordinary least squares linear regression models, we regressed the change in HRQL between baseline and 36 months follow-up on three categories of FEV_1_ change and covariates to calculate mean changes in HRQL. The within-subject change in FEV_1_ after 36 months was defined as either decrease in absolute FEV_1_ ≥ 100 ml, increase in absolute FEV_1_ ≥ 100 ml, and no change (in between). The 100 ml cut-off in FEV_1_ was chosen in accordance with the previously published minimal important difference for COPD [25]. As we considered the change in FEV_1_ to be dependent on baseline lung function, an interaction term to account for the relation between the FEV_1_ change category and baseline FEV_1_ was incorporated.

Second, generalized additive models (GAM) were conducted, to investigate the relationship between HRQL and a continuous measure of FEV_1_. This nonparametric regression models the association between the dependent variable change in HRQL and the independent variable change in FEV_1_ using a smoothing function while adjusting for covariates. Further details have been published elsewhere [26].

#### Hierarchical linear model

We applied hierarchical linear models (HLM), which enable the inclusion of time-variant and time-invariant co-variates and can be applied on datasets with missing variables at different time points (i.e. patient dropped out after second follow-up). These models were designed to provide information regarding mean population trends and individual change over time. Considering time points as time nested in individuals, the model divides the original independent variable into the mean over time (between-subject differences) and the deviation from the mean over time (within-subject change) [27]. In our specific case, the model distinguished between the cross-sectional between-subject and the longitudinal within-subject association of FEV_1_ (included as a continuous variable with the unit 100 ml difference or change) and HRQL.

#### Sensitivity analysis

To account for selective dropout bias, we performed a sensitivity analysis including Inverse Probability Weights (IPW) in the change score- and hierarchical linear models. We first modelled the probability of follow-up based on baseline characteristics (demographics, disease characteristics and quality of life). Weights were then assigned to all patients, who were included in the present analysis, by calculating the inverse of the estimated probability of follow-up. Using this approach, patients, who were found to be similar to those who dropped out, were given greater weights resulting in a weighted population simulating a population without dropout.

All analyses were carried out using the SAS software (SAS Institute Inc., Cary, NC, USA, Version 9.4) package.

## Results

Of the 2741 patients recruited into the COSYCONET cohort, 450 had to be excluded because of missing or non-obstructive spirometry at baseline. Of those entering the cohort (*n* = 2291), 1724 were seen at the second, and 1182 at third follow-up visit. Another 10 participants skipped the first follow-up, but were re-examined in the second follow-up and thus included for the present analysis, resulting in a sample size of *n* = 1734 at baseline.

Table 1 displays the baseline characteristics of the study sample, stratified by GOLD grade 1/2 versus 3/4. Patients with COPD GOLD 1/2 were found slightly older and reported a greater number of comorbidities. The proportion of patients reporting at least one severe exacerbation in the 12 months before the baseline examination was greater for GOLD grade 3/4, as was the proportion of underweight patients. Similarly, mean baseline SGRQ total score and EQ VAS indicated worse HRQL for GOLD 3/4 compared to GOLD 1/2.

**Table 1:** Characteristics of the study population at baseline

		Total sample	GOLD 1/2	GOLD 3/4	
**n**		1734	943	791	*p*-value^1^
Male		1054 (60.8)	570 (60.5)	484 (61.2)	0.7523
Age, yrs		64.6 ± 8.2	65.5 ± 8.3	63.6 ± 8.0	<.0001
Age category	< 55	203 (11.7)	96 (10.2)	107 (13.5)	<.0001
	55–64	610 (35.2)	300 (31.8)	310 (39.2)	
	65–74	750 (43.3)	432 (45.8)	318 (40.2)	
	> = 75	171 (9.9)	115 (12.2)	56 (7.1)	
BMI category^2^	Normal	648 (37.4)	319 (33.8)	329 (41.6)	<.0001
	Overweight	642 (37.0)	366 (38.8)	276 (34.9)	
	Obese	392 (22.6)	242 (25.7)	150 (19.0)	
	Underweight	52 (3.0)	16 (1.7)	36 (4.6)	
FEV_1_ (liters)		1.61 ± 0.64	2.00 ± 0.56	1.13 ± 0.33	<.0001
FEV_1_% predicted		54.1 ± 18.4	69.6 ± 12.8	38.0 ± 8.2	<.0001
Education	Primary	939 (54.2)	480 (50.9)	459 (58.0)	0.0017
	Secondary	487 (28.1)	270 (28.6)	217 (27.4)	
	Higher	308 (17.8)	193 (20.5)	115 (14.5)	
Smoking status	Never smoker	124 (7.2)	73 (7.7)	51 (6.5)	<.0001
	Current smoker	403 (23.2)	258 (27.4)	145 (18.3)	
	Former smoker	1207 (69.6)	612 (64.9)	595 (75.2)	
Comorbidities	Mean number	3.8 (2.6)	3.9 ± 2.6	3.6 ± 2.5	0.0171
Exacerbation history^3^	none	806 (46.5)	519 (55.0)	287 (36.3)	<.0001
	mild	86 (5.0)	57 (6.0)	29 (3.7)	
	moderate	529 (30.5)	266 (28.2)	263 (33.3)	
	severe	313 (18.1)	101 (10.7)	212 (26.8)	
HRQL measures	SGRQ total score	41.6 ± 19.3	35.5 ± 18.5	48.8 ± 17.7	<.0001
	Activity component	56.2 ± 25.5	46.6 ± 24.3	67.6 ± 21.9	<.0001
	Symptoms component	54.4 ± 21.1	50.4 ± 21.6	59.1 ± 19.6	<.0001
	Impacts component	28.6 ± 19.8	23.8 ± 18.7	34.2 ± 19.4	<.0001
	EQ VAS	57.7 ± 19.6	62.9 ± 18.6	51.6 ± 19.0	<.0001

Data are presented as mean ± SD or n (%)

^1^*p*-values based on Chi-square-Tests and ANOVA

^2^BMI groups were defined as normal weight (18.5 ≤ BMI < 25), overweight (25 ≤ BMI < 30), obese (BMI ≥ 30), and underweight (BMI < 18.5)

^3^previous 12 months before examination

*BMI* Body mass index; *FEV*_*1*_ forced expiratory volume in 1 s; *HRQL* Health-related quality of life; *SGRQ* Saint George’s Respiratory Questionnaire; *EQ VAS* Visual Analog Scale

### Change in FEV_1_ and HRQL over three years

For 1182 COPD patients with participation at baseline and at the 36 months follow-up visit, mean change in FEV_1_ and HRQL was calculated (Table 2). Baseline characteristics of this subpopulation are available in Additional file 1. For the 3 years time period, a 150 ml FEV_1_ decrease was observed for all patients, while this decrease was 180 ml for those with GOLD 1/2 and 90 ml for those with GOLD 3/4 at baseline. Over the same period, we also observed a statistically significant deterioration in disease-specific HRQL (SGRQ total score + 1.3 units) on the population level. This overall change in SGRQ was fully driven by a + 4 units change in the activity component, which was present for both baseline GOLD strata. On an individual level, 73% of patients experienced a clinically relevant change in SGRQ after 3 years (40% deterioration, 33% improvement) (Additional file 1 Table A2).

**Table 2:** Change in FEV_1_ and HRQL for 1182 COPD patients who complete the 36-month follow-up

		Baseline	18-month	36-month	3 year change^**1**^	
FEV_1_% predicted	Total sample *[missing values]*	56.1 (18.2) *[−]*	54.4 (18.5) [17]	53.0 (19.2) [9]	−3.0	<.0001
	GOLD 1/2	68.0 (12.8)	65.3 (14.7)	64.1 (15.5)	−3.9	<.0001
	GOLD 3/4	38.8 (8.1)	38.3 (9.8)	37.0 (11.0)	−1.8	<.0001
FEV_1_ (liters)	Total sample	1.68 (0.65) *[−]*	1.60 (0.63) [17]	1.53 (0.64) [9]	−0.15	<.0001
	GOLD 1/2	2.02 (0.57)	1.90 (0.58)	1.84 (0.58)	−0.18	<.0001
	GOLD 3/4	1.17 (0.34)	1.14 (0.38)	1.08 (0.41)	−0.09	<.0001
SGRQ total score	Total sample	40.2 (19.1) [6]	39.7 (20.4) [22]	41.5 (20.4) [17]	1.3	0.0015
	GOLD 1/2	35.0 (18.3)	34.3 (19.3)	35.9 (19.6)	0.9	0.1201
	GOLD 3/4	47.9 (17.7)	47.9 (19.2)	49.8 (18.8)	1.9	0.0019
Activity component	Total sample	54.2 (25.3) [4]	54.8 (26.7) [17]	58.2 (26.9) [16]	4.0	<.0001
	GOLD 1/2	45.9 (24.1)	46.6 (26.0)	49.6 (26.1)	3.7	<.0001
	GOLD 3/4	66.2 (22.0)	67.1 (22.9)	71.0 (22.7)	4.8	<.0001
Symptoms component	Total sample	54.1 (21.3) [3]	52.2 (22.6) [18]	53.4 (22.0) [16]	− 0.7	0.2494
	GOLD 1/2	50.5 (21.6)	48.4 (22.4)	49.4 (22.5)	−1.1	0.1403
	GOLD 3/4	59.4 (19.7)	57.8 (21.6)	59.3 (19.8)	−0.1	0.9660
Impacts component	Total sample	27.2 (19.6) [3]	26.5 (20.3) [19]	27.5 (20.6) [15]	0.3	0.6627
	GOLD 1/2	23.2 (18.6)	22.1 (18.7)	23.0 (19.3)	−0.2	0.5685
	GOLD 3/4	33.1 (19.5)	33.1 (20.9)	34.0 (20.8)	0.9	0.1990
EQ VAS	Total sample	59.1 (19.4) [9]	59.9 (19.5) [22]	58.6 (19.5) [10]	−0.5	0.2830
	GOLD 1/2	63.5 (18.2)	64.1 (18.6)	62.4 (18.7)	−1.1	0.0899
	GOLD 3/4	52.7 (19.4)	53.7 (19.1)	52.9 (19.3)	0.2	0.8051

Data are presented as mean (SD), *[number of missing values]*

Patient numbers in each GOLD group: Total sample *n* = 1182; GOLD1/2 *n* = 702; GOLD 3/4 *n* = 480

^1^p-values based on paired t-test statistics

Analysing the change of the generic EQ VAS in the same way, no significant change was observed on the population level. However, 66% of patients experienced a clinically relevant change in EQ VAS (34% deterioration, 32% improvement).

### Relationship between FEV_1_ and HRQL over time

#### Change score analysis

We then analysed all pairs of repeated FEV_1_ and HRQL measurements stemming from 1173 patients who completed the follow-up after 36 months. Altogether, COPD patients with GOLD 1/2 at baseline contributed 695 pairs of observations, while those with GOLD 3/4 contributed 478 pairs. We observed a ≥ 100 ml FEV_1_ decrease in 58% of the total sample, 28% were recorded as no change in FEV_1_, and the remaining 13% experienced a ≥ 100 ml FEV_1_ increase over the 3 years period (Table 3).

**Table 3:** Change in FEV_1_ over 36 months stratified by baseline GOLD grades

	Change in FEV_**1**_		
	decrease ≥ 100 ml	no change	increase ≥ 100 ml
**GOLD 1/2 (*n*** **= 695)**	63,6%	24,6%	11,8%
**GOLD 3/4 (*n*** **= 478)**	50,4%	33,7%	15,9%
**Total sample (*n*** **= 1173)**	58,2%	28,3%	13,5%
Mean FEV_1_ change	− 311 ml	−11 ml	269 ml
Responder^1^ SGRQ	29,1%	35,5%	48,4%
Responder^1^ VAS	26,5%	37,2%	43,9%

^1^Indicates the percentage of patients who experienced a clinically relevant improvement in HRQL

**Fig. 2:**
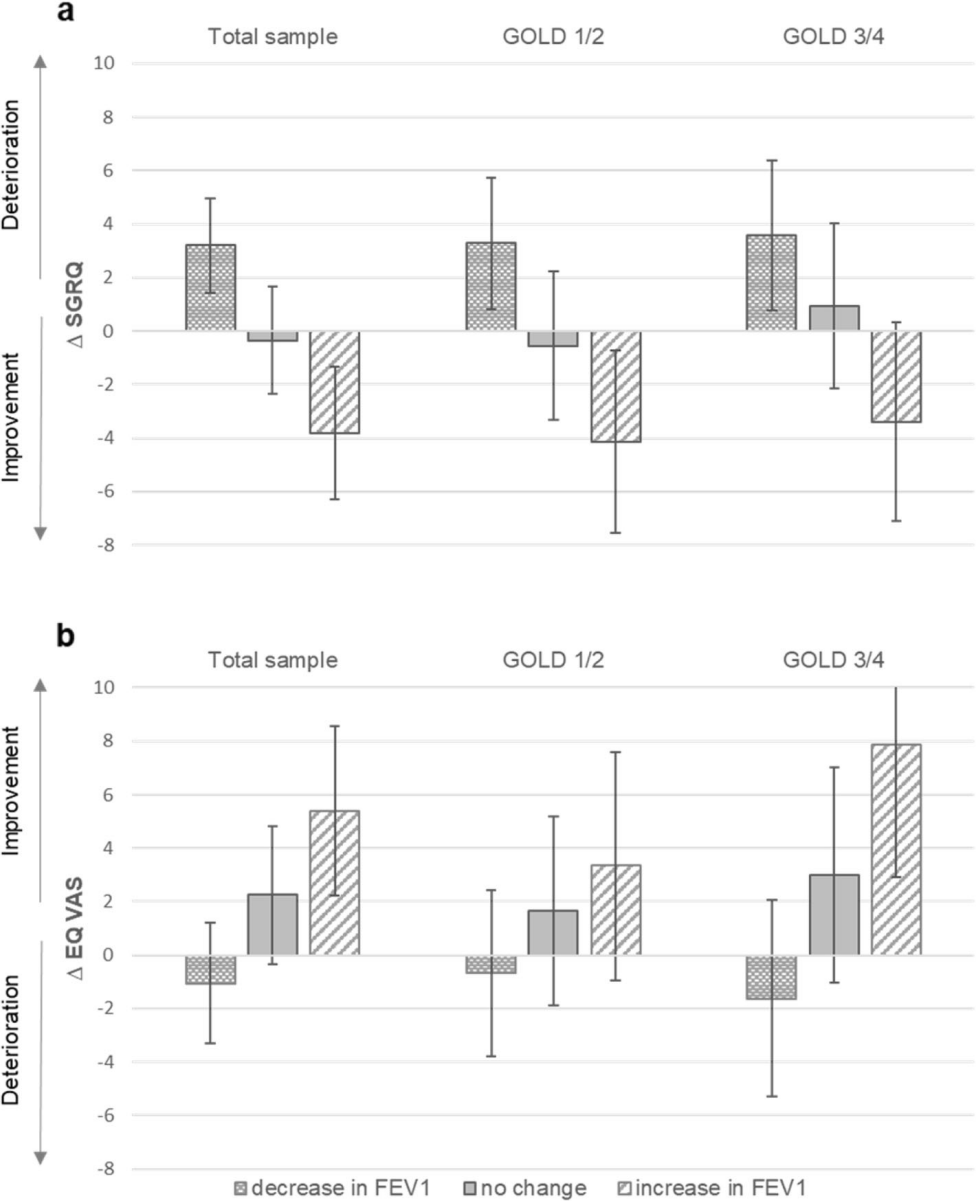
Absolute adjusted mean change in SGRQ (a) and EQ VAS (b) after 36 months. Ordinary least square regression models were adjusted for age, sex, BMI, education, smoking status, number of comorbidities, exacerbation history, and FEV_1_ change*baseline FEV_1_. Error bars indicate 95% confidence intervals. Change categories in FEV_1_ were defined as decrease in absolute FEV_1_ ≥ 100 ml, increase in absolute FEV_1_ ≥ 100 ml, and no change (in between) after 36 months

**Fig. 3:**
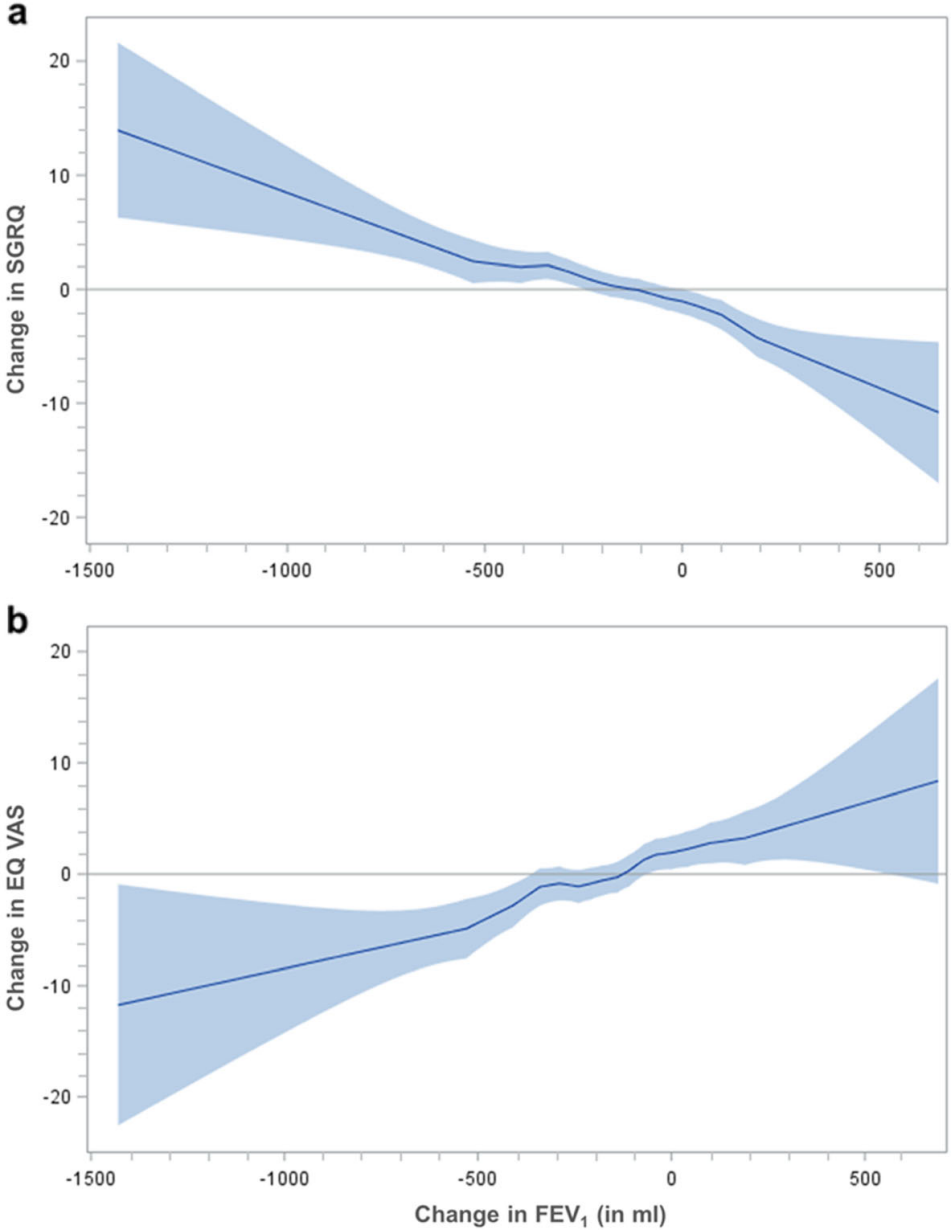
Relationship between change in FEV_1_ and SGRQ (a), EQ VAS (b). Generalized additive models were adjusted for age, sex, BMI, education, smoking status, number of comorbidities, and exacerbation history. The solid curves represent the estimated smooth functions of the association between FEV_1_ and HRQL. The shaded areas indicate 95% confidence intervals

Figure 2 displays the adjusted mean change in SGRQ and EQ VAS as associated with FEV_1_ change (decrease, no change, increase) for all participants and stratified by baseline GOLD grade of severity. Overall, a decrease in FEV_1_ was associated with a deterioration in disease-specific and generic HRQL (mean change [95% CI] SGRQ + 3.20 [1.43 to 4.97], EQ VAS -1.05 [− 3.32 to 1.22]), although this was not significant for EQ VAS. On the other hand, we observed statistically significant improvement in generic and disease-specific HRQL for all patients with increased FEV_1_ (SGRQ -3.81 [− 6.28 to − 1.34], EQ VAS + 5.38 [3.34 to 7.86]). Regarding the category no change in FEV_1_, we found non-significant improvements in EQ VAS while the SGRQ remained unchanged. Both GOLD strata mirrored the results of the total sample. Our data indicated an approximately linear relationship between change in HRQL and FEV_1_ (Fig. 3)_._ However, the graph was found to be shifted to the left side of the x-axis meaning that a zero change in FEV_1_ did not correspond to a zero change in HRQL but was associated with slight improvements in HRQL. Consequently, a clinical relevant deterioration in SGRQ was associated with a decrease in FEV_1_ of more than − 600 ml, while an increase of more than 200 ml FEV_1_ was associated with a clinical relevant improvement in SGRQ.

#### Hierarchical linear model

The results of the HLM analysis detailing the cross-sectional (between-subject) and longitudinal (within-subject) estimates for HRQL and FEV_1_ are presented in Tables 4 and 5. Regarding the SGRQ total score (Table 4) and according to the cross-sectional estimate, higher FEV_1_ was associated with better HRQL with 100 ml more (difference) FEV_1_ corresponding to a mean improvement by − 1.42 units in SGRQ. Corresponding estimates for the single GOLD strata were − 1.00 for GOLD 1/2, and − 1.57 for GOLD 3/4. Conversely, the longitudinal within-subject estimate indicated the effect of a 100 ml FEV_1_ decrease within a patient. Overall, a 100 ml decrease in FEV_1_ resulted in a deterioration in disease-specific HRQL, indicated as a 0.83 units change in SGRQ (*p* < 0.0001).

**Table 4:** Cross-sectional and longitudinal estimates for the association between FEV_1_ and disease-specific HRQL as measured with the SGRQ

***Outcome: SGRQ***	Total sample	GOLD 1/2	GOLD 3/4
	*estimate [95% CI]*	*estimate [95% CI]*	*estimate [95% CI]*
FEV_1_ between-subjects	−1.42* [− 1.55 to − 1.29]	−1.00* [− 1.23 to −0.78]	−1.57* [− 1.95 to − 1.19]
FEV_1_ within-subjects	0.83* [0.65 to 1.01]	0.86* [0.63 to 1.09]	0.92* [0.60 to 1.23]

*p < 0.001

Hierarchical linear models (HLM) adjusted for age, sex, BMI, education, smoking status, number of comorbidities, and exacerbation history

Interpretation: Positive estimates indicate deterioration in HRQL. FEV_1_ between-subjects: cross-sectional difference in HRQL per 100 ml difference in FEV_1_ between subjects. FEV_1_ within-subjects: longitudinal change in HRQL per 100 ml decrease in FEV_1_ within subjects over time

**Table 5:** Cross-sectional and longitudinal estimates for the association between FEV_1_ and generic HRQL as measured with the EQ VAS

***Outcome: EQ VAS***	Total sample	GOLD 1/2	GOLD 3/4
	*estimate [95% CI]*	*estimate [95% CI]*	*estimate [95% CI]*
FEV_1_ between-subjects	1.08* [0.95 to 1.21]	0.71* [0.49 to 0.93]	1.20* [0.83 to 1.58]
FEV_1_ within-subjects	−0.87* [− 1.13 to − 0.62]	−0.67* [− 0.98 to − 0.35]	−1.20* [− 1.64 to − 0.76]

*p < 0.001

Hierarchical linear models (HLM) adjusted for age, sex, BMI, education, smoking status, number of comorbidities, and exacerbation history

Interpretation: Positive estimates indicate improvement in HRQL. FEV_1_ between-subjects: cross-sectional difference in HRQL per 100 ml difference in FEV_1_ between subjects. FEV_1_ within-subjects: longitudinal change in HRQL per 100 ml decrease in FEV_1_ within subjects over time

Regarding the three component scores of the SGRQ, we observe statistically significant longitudinal associations between a 100 ml decrease in FEV_1_ and all domains (activity, symptoms, and impacts). The biggest impact of FEV_1_ decrease was found on the activity domain, followed by the symptoms- and impacts components (data not shown).

Regarding the generic EQ VAS (Table 5), we observed estimates of the same direction, but overall estimates were smaller regarding both the between- and within-subjects analysis in relation to a 100 ml FEV_1_ difference or change, respectively.

#### Sensitivity analysis

The results of the sensitivity analysis are displayed in Additional file 2 (Change Score model) and Additional file 3 (HLM). Overall, the inclusion of IPW confirmed our results since all estimates and *p*-values were nearly identical. However, it also indicated a slight underestimation of the effect of change in FEV_1_ on HRQL particularly in patients with GOLD 3/4 at baseline when excluding dropouts. For example, in patients with baseline GOLD 3/4, the deterioration in SGRQ associated with decrease in FEV_1_ was more pronounced when considering participants who dropped out through IPW (SGRQ mean change + 4.11 [1.37 to 6.84] including IPW vs. + 3.59 [0.79 to 6.38] without IPW (see Additional file 2).

## Discussion

We analysed the change in HRQL over 3 years associated with change in FEV_1_ and investigated both the cross-sectional and the longitudinal association of FEV_1_ and HRQL. On the population level, the overall decline in SGRQ total score was small but statistically significant and was completely driven by a significant deterioration of + 4 units in the activity domain. On the individual patient level, more than one-third of patients experienced a clinically relevant deterioration in SGRQ. We found a linear relationship between change in FEV_1_ and change in HRQL meaning that decrease in FEV_1_ was associated with a deterioration in HRQL whereas an increase in FEV_1_ was similarly found associated with improved HRQL. Remarkably, a no change in FEV_1_ was also associated with slight improvements in HRQL. We found a highly significant relation between a 100 ml within-subject FEV_1_ decrease and generic and disease-specific HRQL, with the largest estimate for the activity domain of the SGRQ.

The overall decline in the disease-specific HRQL in COPD is in line with the literature [12, 28, 29]. Noteworthy enough, the decline was not steady over time, as we found small reductions in the SGRQ total score (i.e. improvement in HRQL) and symptoms component as well as in the EQ VAS for the first 18-months of follow-up, similar to what has been previously observed by Yoo and co-workers [30].

The deterioration in SGRQ was completely driven by the activity component. This aligns with Waschki et al. who reported a substantial decrease in physical activity over 3 years in a COPD cohort [31]. The finding, that the symptoms and impact component remained unchanged or even improved, would be compatible with the assumption that these factors can be managed through adequate medical or non-medical therapy [14]. Our data underline that maintenance of physical activity should play a much greater role in the treatment of COPD.

The mean changes in HRQL as related to the three FEV_1_ change categories did not exceed the MCID. However, a mean deterioration in HRQL on the population level, which is significantly different from zero, indicates an important development, given that a relevant proportion of patients experienced a clinical relevant change in HRQL after 3 years. Furthermore, our results are in line with a systematic review by Westwood and co-workers, who summarized the information of 22 randomized controlled trials on the effects of long-acting bronchodilator therapy and analysed the relationship between increase in FEV_1_ and patient-reported outcomes, including HRQL as measured using the SGRQ [13]. According to this analysis, a mean 2.5 units decrease in SGRQ total score (i.e. improvement) was estimated for a 100 ml increase in FEV_1_.

Our results partly concur with Westwood et al., finding that even no change in FEV_1_ is associated with improved HRQL. While the GAM indicated slight improvements in HRQL for a zero change in FEV_1_, the stratified analysis confirmed this only for patients with baseline GOLD grades 1/2, but indicated a trend for deteriorations in SGRQ for the more severe grades GOLD 3/4. Westwood et al. discuss a potential Hawthorne effect – a phenomenon whereby patients modify their behaviour because of their active participation in science and their awareness of being observed [32]. However, in our study, this effect might be small because the intensity of supervision is rather low with more than a year between study visits. Adaptation processes or changes in treatment after recruitment into the cohort might rather play a role and additional research is needed to further explore this.

The observational Japanese COPD cohort HOKKAIDO evaluated the relationship between FEV_1_ decline and change in SGRQ and its component scores. Based on the degree of the annual decline in FEV_1_, the cohort was split into three categories: rapid decliner (− 63 ± 2 ml/year), slow decliner (− 31 ± 1 ml/year) and sustainers (including improvements in FEV_1_ (− 2 ± 1 ml/year)). The authors report deterioration in HRQL for the rapid decliners indicated by a change of 5 units of the SGRQ total score after 5 years, zero change for slow decliners and an improvement in HRQL (− 4 units SGRQ) for the sustainers [14]. Calculation of the change in SGRQ per 100 ml FEV_1_ decrease based on the data given for the rapid decliner, results in a mean deterioration in HRQL by a 1.59 units change in SGRQ total score. The within-subject estimate of our HLM indicated a deterioration in HRQL of half the size (+ 0.83 units SGRQ per 100 ml FEV_1_ decrease), which is not surprising, considering that our population was not stratified by categories of FEV_1_ decline.

Both HRQL measures differentiated between GOLD strata at baseline and the longitudinal within-subject association between FEV_1_ and HRQL showed a similar relationship. However, the overall change in EQ VAS after 3 years (− 0.5 units, n.s.) might have been too small to detect significant mean changes in EQ VAS as related to the FEV_1_ change category decrease. Methodological aspects could explain part of the observed differences. Whereas the SGRQ covers history and current health status, the EQ VAS refers to the patients’ current short-term health status, which might show more variation than a sort of averaging as implemented in the SGRQ. Moreover, the EQ VAS as a generic measure of HRQL includes aspects of the patients’ life that are not related to his/her COPD all. We conclude that disease-specific instruments are more suitable for the longitudinal assessment of HRQL in patients with COPD.

Selective dropout of patients is an issue in long-term observational cohort studies. Regarding our data set of 2291 eligible patients recruited at baseline of the COSYCONET cohort study, 557 and 552 patients were not re-examined at the 18-month and 36-month follow-up visit, respectively. Of those 1109 patients, 153 (14%) died and 296 (27%) terminated their participation due to worsening of their health status. However, we do not think that dropout severely affected our findings and the sensitivity analysis including the IPW confirmed this hypothesis. One reason might be that our aim was to analyse the association of change in FEV_1_ and HRQL and not to predict HRQL development. The latter would indeed be influenced by dropout as one would expect those with deteriorating COPD to also experience worse HRQL. Second, the hierarchical linear model also included patients who were available for only two examinations, therefore minimizing the number of patients not considered.

With regard to the observational and longitudinal design of our study, some limitations need to be addressed. First, regression to the mean might have occurred in the repeated measurement of lung function and HRQL values [33]. This bias seems, however, unlikely since we were interested in the association between the change in FEV_1_ and the change in HRQL, which was independent from FEV_1_ group assignment. Furthermore, longitudinal results were also confirmed by the HLMs, which are thought to be robust against a bias from regression to the mean. Second, our analyses do not allow drawing conclusions regarding treatment effects on lung function. All patients were under their usual therapy, but medication-specific variables were not considered in the models. This aspect might, however, be less important, as in general the treatment in the COSYCONET cohort is very intense and broad [34].

## Conclusions

To conclude, our study provides estimates for both the cross-sectional and longitudinal association between FEV_1_ and HRQL and these were highly statistically significant regarding both outcomes: disease-specific and generic HRQL. Overall, change in HRQL followed change in FEV_1_, however, increases in FEV_1_ were associated with greater HRQL gains than equal decreases in FEV_1_ with HRQL losses. To monitor the progression of COPD from the patient’s perspective, the disease-specific SGRQ was found superior to the generic EQ-VAS. As quality of life is an important aspect in patients’ life, determining the course of the disease and therapeutic requirements, the findings suggest that optimal treatment of lung function and a minimization of its deterioration over time has an impact beyond the patients’ functional status. Furthermore, deterioration of HRQL should induce timely re-examination of physical status and lung function and possibly reassessment of therapeutic regimes, particularly in patients with severe airflow obstruction.

## Supplementary information

**Additional file 1.** Table A1 Baseline characteristics of COPD patients who completed the 36-month follow-up and Table A2 Clinically important change in HRQL after 36-month.

**Additional file 2.** Inverse Probability Weighting: Absolute adjusted mean change in SGRQ (a) and EQ VAS (b) after 36 months

**Additional file 3.** Table A3 Inverse Probability Weighting: Cross-sectional and longitudinal estimates for the association between FEV1 and disease-specific HRQL as measured with the SGRQ.

## Data Availability

Data may be obtained from a third party and are not publicly available. The full dataset supporting the conclusions of this article is available upon request and application from the Competence Network Asthma and COPD (ASCONET, http://www.asconet.net/html/cosyconet/projects).

## Abbreviations

BMI: Body mass index
COPD: Chronic obstructive pulmonary disease
COSYCONET: COPD and systemic consequences - comorbidities network
EQVAS: Euro-qol visual analog scale
EQ-5D: Euro-qol 5 dimensions questionnaire
FEV_1_: Forced expiratory volume in 1 s
FVC: Forced vital capacity
GAM: Generalized additive model
GOLD: Global Initiative for chronic obstructive pulmonary disease
HLM: Hierarchical linear model
HRQL: Health-related quality of life
IPW: Inverse probability weights
RCT: Randomized controlled trial
SGRQ: Saint George’s respiratory questionnaire
