# Supplementary material for: Health-related quality of life associates with change in FEV1 in COPD: results from the COSYCONET cohort
Source: BMC Pulm Med. 2020 May 29;20:148. doi: 10.1186/s12890-020-1147-5 (PMC7257512; doi:10.1186/s12890-020-1147-5)
Supplement: Supplementary file 1 — Additional file 1. Table A1 Baseline characteristics of COPD patients who completed the 36-month follow-up and Table A2 Clinically important change in HRQL after 36-month. [file 12890_2020_1147_MOESM1_ESM.docx]

**Additional file 1**

**Table A1 Baseline characteristics of COPD patients who completed the 36-month follow-up**

|  |  | **Total sample** | **GOLD 1/2** | **GOLD 3/4** |  |
| --- | --- | --- | --- | --- | --- |
| **n** |  | 1182 | 702 | 480 | p-value^1^ |
| Male |  | 719 (60.8) | 424 (60.4) | 295 (61.5) | 0.7140 |
| Age, yrs |  | 64.4 ± 8.2 | 65.5 ± 8.3 | 63.6 ± 8.0 | <.0001 |
| Age category | < 55 | 144 (12.2) | 73 (10.4) | 71 (14.8) | <.0001 |
|  | 55 - 64 | 427 (36.1) | 227 (32.3) | 200 (41.7) |  |
|  | 65-74 | 503 (42.6) | 323 (46.0) | 180 (37.5) |  |
|  | >= 75 | 108 (9.1) | 79 (11.3) | 29 (6.0) |  |
| BMI category^2^ | Normal | 421 (35.6) | 224 (31.9) | 197 (41.0) | 0.0002 |
|  | Overweight | 462 (39.1) | 284 (40.5) | 178 (37.1) |  |
|  | Obese | 269 (22.8) | 182 (25.9) | 87 (18.1) |  |
|  | Underweight | 30 (2.5) | 12 (1.7) | 18 (3.8) |  |
| FEV_1_ (liters) |  | 1.68 ± 0.65 | 2.02 ± 0.57 | 1.17 ± 0.34 | <.0001 |
| FEV_1_% predicted |  | 56.1 ± 18.2 | 68.0 ± 12.8 | 38.8 ± 8.1 | <.0001 |
| Education | Primary | 619 (52.4) | 345 (49.2) | 274 (57.1) | 0.0156 |
|  | Secondary | 336 (28.4) | 207 (29.5) | 129 (26.9) |  |
|  | Higher | 227 (19.2) | 150 (21.4) | 77 (16.0) |  |
| Smoking status | Never smoker | 86 (7.3) | 52 (7.4) | 34 (7.1) | 0.0003 |
|  | Current smoker | 273 (23.1) | 190 (27.1) | 83 (17.3) |  |
|  | Former smoker | 823 (69.6) | 460 (65.5) | 363 (75.6) |  |
| Comorbidities | Mean number | 3.8 ± 2.6 | 3.9 ± 2.6 | 3.6 ± 2.6 | 0.0494 |
| Exacerbation history^3^ | none | 557 (47.1) | 388 (55.3) | 169 (35.2) | <.0001 |
|  | mild | 60 (5.1) | 39 (5.6) | 21 (4.4) |  |
|  | moderate | 369 (31.2) | 201 (28.6) | 168 (35.0) |  |
|  | severe | 196 (16.6) | 74 (10.5) | 122 (25.4) |  |
| HRQL measures | SGRQ total score | 40.2 ± 19.1 | 35.0 ± 18.3 | 47.9 ± 17.7 | <.0001 |
|  | Activity | 54.2 ± 25.3 | 45.9 ± 24.1 | 66.2 ± 22.0 | <.0001 |
|  | Symptoms | 54.1 ± 21.3 | 50.5 ± 21.6 | 59.4 ± 19.7 | <.0001 |
|  | Impacts | 27.2 ± 19.6 | 23.2 ± 18.6 | 33.1 ± 19.5 | <.0001 |
|  | EQ VAS | 59.1 ± 19.4 | 63.5 ± 18.2 | 52.7 ± 19.4 | <.0001 |

Data are presented as mean ± SD or n (%)

^1^ p-values based on Chi-square-Tests and ANOVA

^2^ BMI groups were defined as normal weight (18.5 ≤ BMI <25), overweight (25 ≤ BMI < 30), obese (BMI ≥ 30), and underweight (BMI < 18.5).

^3^ previous 12 months before examination

**Table A2 Clinically important change in HRQL after 36-month**

| **SGRQ** | **Total sample** | **GOLD 1/2** | **GOLD 3/4** |
| --- | --- | --- | --- |
| n | 1159^1^ | 690 | 469 |
| clinically important deterioration | 458 (39,5) | 260 (37,7) | 198 (42,2) |
| No change | 314 (27,1) | 191 (27,7) | 123 (26,2) |
| clinically important improvement | 387 (33,4) | 239 (34,6) | 148 (31,6) |
| **VAS** | **Total sample** | **GOLD 1/2** | **GOLD 3/4** |
| n | 1163^2^ | 692 | 471 |
| clinically important deterioration | 398 (34,2) | 230 (33,2) | 168 (35,7) |
| No change | 399 (34,3) | 258 (37,3) | 141 (29,9) |
| clinically important improvement | 366 (31,5) | 204 (29,5) | 162 (34,4) |

Data are presented as n (%)
