# Supplementary material for: Health-related quality of life associates with change in FEV1 in COPD: results from the COSYCONET cohort
Source: BMC Pulm Med. 2020 May 29;20:148. doi: 10.1186/s12890-020-1147-5 (PMC7257512; doi:10.1186/s12890-020-1147-5)
Supplement: Supplementary file 2 — Additional file 2. Inverse Probability Weighting: Absolute adjusted mean change in SGRQ (a) and EQ VAS (b) after 36 months [file 12890_2020_1147_MOESM2_ESM.docx]

**Additional file 2**

**Inverse Probability Weighting:** Absolute adjusted mean change in SGRQ (a) and EQ VAS (b) after 36 months

**
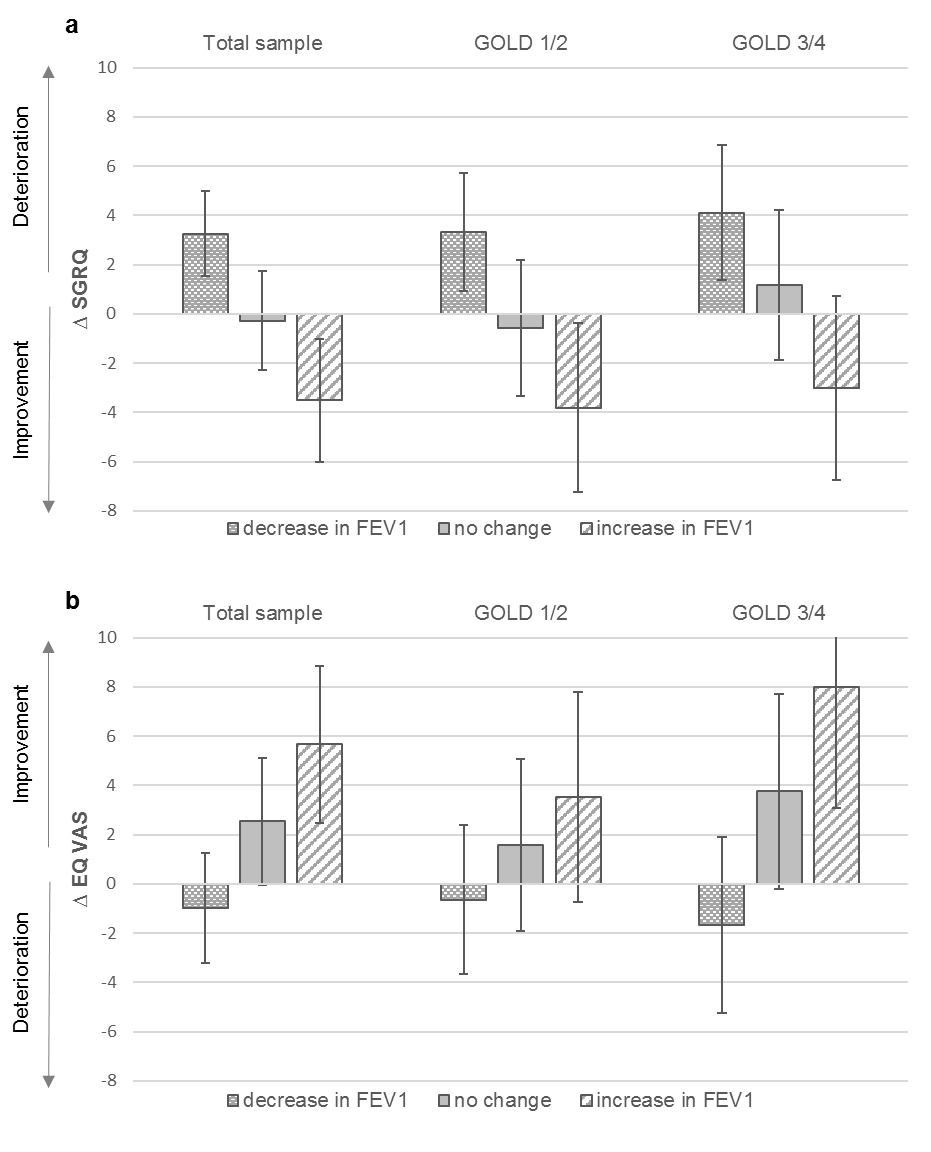
**

Ordinary least square regression models were adjusted for age, sex, BMI, education, smoking status, comorbidity burden, exacerbation history, and FEV_1_ change*baseline FEV_1_. Error bars indicate 95% confidence intervals. Models include inverse probability weights to account for dropout.
Change categories in FEV_1_ were defined as decrease in absolute FEV_1_ ≥ 100 ml increase in absolute FEV_1_ ≥ 100 ml, and no change (in between) after 36 months.
