# Supplementary material for: Health-related quality of life associates with change in FEV1 in COPD: results from the COSYCONET cohort
Source: BMC Pulm Med. 2020 May 29;20:148. doi: 10.1186/s12890-020-1147-5 (PMC7257512; doi:10.1186/s12890-020-1147-5)
Supplement: Supplementary file 3 — Additional file 3. Table A3 Inverse Probability Weighting: Cross-sectional and longitudinal estimates for the association between FEV1 and disease-specific HRQL as measured with the SGRQ. [file 12890_2020_1147_MOESM3_ESM.docx]

**Additional file 3**

**Table A3**

**Inverse Probability Weighting:** Cross-sectional and longitudinal estimates for the association between FEV_1_ and disease-specific HRQL

| ***Outcome: SGRQ*** | **Total sample** | **GOLD 1/2** | **GOLD 3/4** |
| --- | --- | --- | --- |
|  | *estimate [95 % CI]* | *estimate [95 % CI]* | *estimate [95 % CI]* |
| FEV_1_ between-subjects | -1.41***  [-1.54 to -1.27] | -0.99***  [-1.21 to -0.76] | -1.57***  [-1.95 to -1.19] |
| FEV_1_ within-subjects | 0.85***  [0.66 to 1.04] | 0.87***  [0.64 to 1.10] | 0.96***  [0.63 to 1.29] |

*** p < 0.001

Hierarchical linear models (HLM) adjusted for age, sex, BMI, education, smoking status, number of comorbidities, and exacerbation history. Models include inverse probability weights to account for dropout.

Interpretation: Positive estimates indicate deterioration in SGRQ. FEV_1_ between-subjects: cross-sectional difference in HRQL per 100 ml difference in FEV_1_ between subjects. FEV_1_ within-subjects: longitudinal change in HRQL per 100 ml decrease in FEV_1_ within subjects over time.
